# Review of Feeding Systems Affecting Production, Carcass Attributes, and Meat Quality of Ovine and Caprine Species

**DOI:** 10.3390/life13051215

**Published:** 2023-05-19

**Authors:** Tiantian Ke, Mengyu Zhao, Xiaoan Zhang, Yao Cheng, Yiming Sun, Penghui Wang, Chunhuan Ren, Xiao Cheng, Zijun Zhang, Yafeng Huang

**Affiliations:** 1 College of Animal Science and Technology, Anhui Agricultural University, Hefei 230036, China; 2 Yingshang Agricultural Green Development Promotion Center, Yingshang 236200, China; 3 Center of Agriculture Technology Cooperation and Promotion of Dingyuan County, Dingyuan 233200, China

**Keywords:** small ruminants, feeding systems, growth rate, carcass attributes, meat quality

## Abstract

Growth rate, carcass attributes, and meat quality traits of small ruminants (i.e., sheep and goats) depend on various factors, among which the feeding system is one of the most important factors. However, how feeding systems affect these parameters differ between sheep and goats. Therefore, this review aimed to evaluate the differences in how different feeding systems affect the growth performance, carcass characteristics, and meat quality of sheep and goats. It also explored the effects of a new finishing strategy—time-limited grazing with supplements on these traits. Compared with stalled feeding, finishing lambs/kids on pasture-only feed reduced the average daily gain (ADG) and carcass yield, while supplemented-grazing lambs/kids had near-equivalent or higher ADG and carcass attributes. Pasture-grazing increased the meat flavor intensity and healthy fatty acid content (HFAC) of lamb/kid meat. Supplemental grazing lambs had comparable or superior meat sensory attributes and increased meat protein and HFAC compared to stall-fed ones. In contrast, supplemental grazing only improved the meat color of kids but had little effect on other meat qualities. Moreover, time-limited grazing with supplemental concentrates increased the carcass yield and meat quality in lamb meat. Overall, the effects of different feeding systems on growth performance and carcass traits were comparable between sheep and goats but differed in terms of the meat quality.

## 1. Introduction

Driven by the socio-economic development and improvement of consumer health consciousness, the global demand for small ruminant (i.e., sheep and goats) meat products has increased due to their richness in protein, the fact that they contain vitamin B12, and favorable fatty acid (FA) composition [1,2]. The production of small ruminant meat has steadily increased globally from 11.28 to 16.03 million tons between 2000 and 2020 [3]. Furthermore, consumer demands are focused not only on the quantity of sheep/goat meat products, but also on the organoleptic quality and nutritional value of meat, especially in developed countries [4,5,6]. Studies showed that the carcass and meat quality of lambs and goat kids depends on many factors, among which the feeding systems are the most important [7,8], because they directly affects meat quality of lambs and kids [9,10]. Zervas et al. [9] also reported that the feeding system implemented by sheep farmers is important in determining the quality of the small ruminant products produced, influencing the decisions of consumers. This is because consumers will reject these meat products if they are not satisfied with the visual and organoleptic properties and the traits of safety and health of the meat [11].

Feeding systems, including pasture/grassland grazing, stall-fed/concentrate-fed, and grazing plus supplementation, are utilized worldwide for raising small ruminants. Several studies have showed that, compared with stall-fed sheep/goats, pasture-only grazing is cheaper and more practical for producing enriched meat with higher contents of beneficial FAs for human nutrition (e.g., monounsaturated FAs (MUFA), polyunsaturated FAs (PUFA), and n-3 PUFA [12,13,14], antioxidants [15,16], and improved the PUFA/SFA and n-6/n-3 ratios [17]). The production system is also considered more animal welfare-friendly [18]. However, compared with concentrate feeding, grazing on pasture only slows growth rates, leading to carcasses [19,20], and thus reduced efficiency of production [21,22,23], meat tenderness and juiciness, and increased intensity of meat flavor in lambs [24,25,26]. Moreover, the meat from supplemented grazing sheep lambs has a higher protein content and beneficial FA contents compared to concentrate-finished lambs [27]. However, the protein content and FA composition in the *longissimus* muscle of grazed goat kids with concentrate supplementation are comparable to those of stall-fed kids [28,29]. These results suggest that the same feeding system has different effects on the meat quality of sheep and goats. Thus, the differences in the effects of the same feeding strategy on the growth rate, carcass attributes, and meat quality of sheep and goats need to be clarified. In addition, grazing on extensive grasslands (shrublands) is often unavoidable because it is the only resource available to the local population, and few other agricultural activities can be sustained in this environment. However, grazing on natural grasslands, particularly in arid and semi-arid regions, leads to degradation and land desertification due to overgrazing [30]. Therefore, a new system of fattening needs to be developed to eliminate the contradiction between growth production and meat quality and to reduce overgrazing of grasslands. Wang et al. [27,31] revealed that combining time-limited grazing (4 h) and stall-feeding could yield desirable outcomes, including safe and high-quality lamb meat.

Although feeding regimens significantly affect the growth rate, carcass attributes, and meat quality of small ruminants, to date, no systematic review on how or whether a difference exists in the effects of feeding systems on the above parameters in sheep and goats has been published. Moreover, the price of these ruminants depends mainly on the quality of their meat and carcasses. This review describes and evaluates how different feeding systems regulate the growth rates, carcasses, organoleptic quality, and nutritional quality attributes of sheep and goat products. The review also summarizes the current research progress on the new finishing strategy—time-limited grazing combined with stall-feeding.

## 2. Materials and Methods

To aid the creation of the present paper, we performed a narrative review of the original published papers on the effects of feeding systems on the growth performance, carcass attributes, and meat quality of Ovine and Caprine Species. The articles and papers were obtained from Science Direct, PubMed, Google Scholar, Web of Science, and CNKI databases. The search language was English or Chinese. Two reviewers separately searched the literature by title/abstract and selected eligible articles on the effects of feeding systems on growth performance, carcass attributes, and meat quality of sheep or/and goats. Studies that did not meet the eligibility criteria were excluded. The selected articles were scrutinized for their eligibility and data extraction.

## 3. Feeding Systems of Small Ruminants Affect the Growth Rate and Carcass Attributes

Carcass weight and yield are often used nationally or internationally as quantitative or even qualitative indicators for commercial transactions [32], and these attributes determine the price of small ruminants, directly affecting the farmers’ income [33]. Feeding systems greatly influence the growth rate and carcass attributes of sheep and goats (Table 1). Regarding sheep lambs, grazing on natural pasture reduced the average daily gain (ADG), slaughter live weight (SLW), hot carcass weight (HCW), cold carcass weight (CCW), and dressing percentage (DP) of lambs compared with exclusive concentrate diets [4,7,34,35,36,37,38,39]. These effects could be associated with lower forage mass intake and higher energy demand for maneuvering the grazing system [40,41,42,43], a higher digestibility of concentrate diets [44], lower gastrointestinal content of concentrates [45,46] and high or low adaptation of the breed to the feeding environment. However, comparable SLW, CCW, and ADG have been reported for alfalfa (*Medicago sativa* L.) grazing and concentrate-feeding lambs [24]. Notably, Hamdi et al. [47] reported that grazing lambs on natural rangeland improved with lucerne (*Medicago arborea*), increasing the SLW and ADG compared with stall-feeding. However, HCW, CCW, and DP were comparable between the two groups [47]. The differences in the mentioned parameters could be attributed to the high nitrogen content of lucerne, which increases the digestion rate [44,48,49,50] and, as a result, growth. The reviewed findings indicate that improving the quality of natural grassland or establishing artificial grassland could increase the growth rate and carcass yield of lambs. Furthermore, lambs grazing on natural pastures supplemented with concentrates have increased ADG compared to those fed on concentrates [31,37,39]. However, SLW, HCW, CCW, and DP are comparable between the two feeding modes [31,37,39]. These findings suggest that concentrate supplements increase the growth rate and improve the carcass characteristics of grazed lambs. Moreover, time-limited grazing (4 h) with supplementary concentrates increased the HCW of lambs more than concentrate feeding, but had no effect on the ADG [31].

Regarding goat kids, 3-month-old kids grazing on natural pastures have lower HCW, CCW, and DP compared with stall-feeding kids [51], possibly due to the available quantity and quality of pasture and environmental factors (sunshine, rainfall, and dirt) [4]. Diseases from free-range feeding could slow growth and reduce the DP. Additionally, irrigated pasture-only grazing reduced the ADG and SLW of kids compared with stall-fed irrigated pasture [51], possibly because walking consumes energy available for growth under pasture grazing. Wang et al. [52] confirmed that kids grazing on natural pasture showed similar ADG but lower HCW, CCW, and DP compared to those subjected to a mixed stall-fed diet based on alfalfa hay. Furthermore, compared with the concentrate diet, the SLW was heavier under grazing supplemented with concentrates [53]. However, the HCW and DP were comparable between the groups [53]. These results are partly explained by the fact that goats reared on supplementary grazing exercise enough and eat more forage and concentrate, which increases their appetite, thus promoting growth. Moniruzzaman et al. [54] reported that goats fed on time-limited grazing natural pastures had similar SLW but lower HCW and DP than those under stall-feeding conditions. This could be because reduced grazing time could reduce energy loss from exercise but provides an insufficient supplementary diet regarding energy supply for lamb growth. Unfortunately, there are no additional reports on the effects of time-limited grazing and supplementary feeding on the growth rate, carcass attributes, and *longissimus* quality of goat kids.

Overall, compared with concentrate-fed or stall-fed sheep/goats, lambs/kids finished on pasture have inferior ADG and carcass yield. However, lambs/kids grazed on natural pastures plus supplements or improved pastures have comparable or superior ADG and carcass yield than their concentrate-fed counterparts. Time-limited grazing plus supplements increased the HCW of lambs compared with concentrate-finishing, but had little effect on ADG.

**Table 1:** Effects of feeding systems on growth rate and carcass attributes of small ruminants.

Items	Animals	Animals ^1^/Duration (Day)	Treatments	Zootechnical Performances ^2^	References
	2-month-old male Polish Merino lambs	18/60	Concentrate-fed, natural pasture grazing	↓SLW, CCW, ADG	[4]
Sheep	5.7-month-old male Norduz lambs	15/84	Concentrate-fed, natural pasture grazing	↓SLW, HCW, CCW, DP	[7]
	2-month-old male Romane lambs	12/74	Concentrate-fed, alfalfa grassland grazing	=SLW, CCW, ADG	[24]
	Newborn Churra Tensina light lambs	19/—	Concentrate-fed, permanent pasture grazing	↓SLW, HCW, DP	[34]
	3-month-old Sunit sheep	10/270	Stall-feeding, natural pasture grazing	↓SLW, HCW	[35]
	4-month-old female Hulunbui lambs	22/120	Concentrate-fed, pasture grazing	↓SLW, HCW, DP	[36]
	3-month-old Mongolia sheep	10/270	Concentrate-fed, natural pasture grazing	↓SLW, HCW	[38]
	6-month-old male Barbarine lambs	6/90	Stall-feeding, grazing natural pastures improved by lucerne	=HCW, CCW, DP; ↑SLW, ADG	[47]
	3-month-old male Tan lambs	10/120	Concentrate-fed, time-limited grazing natural grassland supplemented with concentrate, grazing natural grassland supplemented with concentrate	=ADG; ↑HCW with time-limited grazing grassland plus supplementary ↑ADG, HCW with supplemental grazing	[31]
	2-month-old Texel lambs	6/104	Concentrate-fed, *Brachiaria* pasture grazing, grazing *Brachiaria* pastures supplemented with concentrate	↓SLW, HCW, CCW, DP with pasture grazing; =SLW, HCW, CCW, DP with supplemental grazing	[39]
	6-month-old male Kheri lambs	20/90	Concentrate-fed, pasture grazing, grazing pastures supplemented with concentrate	↓ADG, SLW, HCW, DP with pasture grazing; =SLW, HCW, DP; ↑ADG with supplemental grazing	[37]
	6-month-old male Barbarine lambs	12/240	Concentrate-fed, grazing native pastures supplemented concentrates	=SLW, HCW, CCW, ADG; ↓DP	[55]
Goats	about 12-month-old Black Bengal goat	6/219	Stall-feeding, time-limited grazing natural pasture, natural pasture grazing	=SLW; ↓HCW, DP with time-limited grazing; =SLW; ↓HCW, DP with pasture grazing	[51]
	3 month-old goats	16/12	Concentrate-fed, natural pasture grazing	=ADG; ↓HCW, CCW, DP	[52]
	4 month-old Albas White Cashmere kids	30/60	Concentrate-fed, natural pasture grazing	↓ADG	[56]
	2.9 month-old male Creole kids	60/—	Stall-feeding, irrigated pasture grazing	=HCW, DP; ↓SLW, ADG	[53]
	3 month-old Chongming white goat	6/300	Concentrate-fed, grazing pastures supplemented with concentrate	=HCW; ↑SLW	[7]

= No effect means that no differences were detected among treatments groups (*p* > 0.05). ↑↓ Positive or negative effect means that the treatments showed differences compared to the concentrate-fed/stall-fed group (*p* < 0.05). ^1^ Number of animals per each treatment groups. ^2^ ADG, average daily gain; CCW, cold carcass weight; DP, dressing percentage; HCW, hot carcass weight; SLW, slaughter live weight; —, none reported.

## 4. Feeding Systems of Small Ruminants Affect Organoleptic Quality Attributes of Meat

Color, tenderness, juiciness, and flavor are the important quality determinants of organoleptic perception of meat [57,58], of which meat color commonly affects the meat quality perception by consumers [59]. Tenderness, juiciness, and flavor are other important characteristics that influence consumer satisfaction [60,61].

### 4.1. Meat Color of Longissimus Muscle

The finishing system has a marked effect on meat color, including lightness (L*), redness (a*), and yellowness (b*) (Table 2), thus impacting consumer preferences [62,63]. Regarding sheep lambs, compared with concentrate-fed lambs, the *longissimus* muscle of the pasture-fed lambs has a lower L*, but comparable or lower a* and b* [7,27,36]. This indicates that concentrate-based diets lead to desirable attributes of *longissimus* for lamb meat. These results could be attributed to changes in reflexivity caused by high-fat deposition in animals fed on concentrate diets, more physical activity, and lower energy intake, resulting in lower SLW under grazing conditions [7,64,65]. High a* is associated with high pigmentation resulting from higher muscle activity and SLW of lambs [7,64]. However, the meat from lambs grazing on natural grassland containing lucerne or grazing exclusively on alfalfa or grazing plus feeding supplements had a similar meat color to stall-fed lambs [24,27,47,55,66]. This may be related to similar growth rates of lambs under these feeding systems [67], indicating that concentrate supplements or grazing on high-quality pastures impart desirable color to lamb meat. Furthermore, Wang et al. [27] reported that the *longissimus* muscle of lambs grazing for 4 h and receiving concentrate supplementation had comparable L* and a* values but a lower b* value than lambs subjected to concentrate diets. This indicated that lamb meat freshness was higher under time-limited grazing with concentrate supplementation than under concentrate feeding because the b* value negatively correlated with meat freshness.

Regarding goat kids, Alexandre et al. [56] found that, compared with stall-fed goat kids, those grazing on irrigated pastures had a lower a* value of *longissimus* muscle. However, the L* and b* values of *longissimus* muscle were comparable between the two groups [56], and their effects could partly increase gastrointestinal parasitism in the pasture-fed kids [68]. The L*, a*, and b* values were higher in the *longissimus* muscle of supplemented grazing kids than those that are fed concentrates [53]. This could be due to higher SLW and lower muscle activity among the stall-fed kids because a higher a* value in kid meat was associated with higher pigmentation due to the higher SLW and muscle activity of kids [7,69]. Therefore, the results of the above study indicate that kids grazed with supplementation can obtain the meat color that consumers desire.

Overall, compared with stall-feeding production systems, pasture-based production systems exhibited less desirable meat color among lambs and kids. In contrast, lambs grazing on pastures plus supplementation or improved pastures had a similar meat color to stall-fed lambs. The meat color of the supplemented grazing kids appealed more to the eye than those fed on concentrates. Time-limited grazing with supplementary feeding increased the lamb meat freshness more than the concentrate feeding.

**Table 2:** Effects of feeding systems on the meat color of small ruminants.

Items	Animals	Animals ^1^/Duration (Day)	Treatments	Histologic Tissue	Zootechnical Performances ^2^	References
Sheep	5.7-month-old male Norduz lambs	15/84	Concentrate-fed, natural pasture grazing	*Longissimus thoracis*	↓L*, b*; =a*	[7]
	4-month-old Hulunbui female lambs	22/120	Concentrate-fed, pasture grazing	*Longissimus dorsi*	↓L*, a*; =b*	[36]
	2-month-old male Romane lambs	12/74	Concentrate-fed, alfalfa grassland grazing	*Longissimus thoracis et lumborum*	=L*, a*, b*	[24]
	6-month-old male Barbarine lambs	6/90	Stall-feeding, grazing natural pastures improved by lucerne	*Longissimus lumborum*	=L*, a*, b*	[47]
	3-month-old male Tan lambs	10/120	Concentrate-fed, natural grassland grazing, time-limited grazing natural grassland supplemented with concentrate, grazing natural grassland supplemented with concentrate	*Longissimus thoracis*	↓L*, b*; =a* with pasture grazing; =L*, a*, ↓ b* time-limited grazing grassland plus supplementary; =L*, a*, b* with supplemental grazing	[27]
	6-month-old male Barbarine lambs	12/240	Concentrate-fed, grazing native pastures supplemented concentrates	*Longissimus thoracis*	=L*, a*, b*	[55]
	3-month-old male Chios lamb	17/60	Concentrate-fed, grazing under olive trees plus supplementation	*Longissimus dorsi*	=L*, a*, b*	[66]
Goats	2.9-month-old male Creole goats	60/—	Stall-feeding, irrigated pasture grazing	*Longissimus*	=L*, b*; ↓a*	[56]
	3-month-old Chongming white goats	6/300	Concentrate-fed, grazing pastures supplemented with concentrate	*Longissimus dorsi*	↑L*, a*, b*	[53]

= No effect means that no differences were detected among treatments groups (*p* > 0.05). ↑↓ Positive or negative effect means that the treatments showed differences compared to the concentrate-fed/stall-fed group (*p* < 0.05). ^1^ Number of animals per each treatment groups. ^2^ L*, lightness; a*, redness; b*, yellowness; —, none reported.

### 4.2. Meat Tenderness and Juiciness of Longissimus Muscle

Feeding systems also markedly affect the meat tenderness and juiciness of small ruminants (Table 3). In fact, meat tenderness is negatively correlated with the Warner-Bratzler shear force (WBSF), which depends on several factors, including ultimate pH, intramuscular fat (IMF), collagen contents, and muscle fiber length [70]. Meat juiciness has a strong positive correlation with water-holding capacity (WHC) and a negative correlation with drip loss (DL), water loss (WL), and cooking loss (CL) [36,70,71].

Studies have showed that, compared with concentrate-fed lambs, the meat of pasture-fed lambs is less tender and juicy, indicated by significantly higher WBSF and lower WHC or higher CL and DL, respectively [36,70,71,72,73]. Concentrates enhance the tenderness and juiciness of lamb meat by reducing the fiber content in the muscles, strength of connective tissue [74], and disintegrating myofibrillar proteins and connective tissue, all of which increase the WHC of proteins [75]. However, grazing lambs on natural rangeland improved by lucerne increased the tenderness and juiciness of meat better than stall-feeding [47], indicating that grazing on high-quality pastures improves the tenderness and juiciness of the meat. Moreover, supplemented grazing lambs had similar meat tenderness and juiciness compared with concentrate-fed animals [27,39,66], possibly due to similar fatness between the two groups, which has a direct positive correlation with tenderness and juiciness [73,76]. Wang et al. [27] reported similar juiciness (as indicated by similar DL values) in the *longissimus* muscle of lambs under time-limited grazing with supplementary feeding compared to animals that are intensively fed concentrates.

The *longissimus* muscle of kids grazing on natural pastures was tenderer and less juicy, as indicated by a significantly lower WBSF value and higher WL value than those of lambs fed on concentrates [77]. This indicated a greater tenderness and reduced juiciness in the *longissimus* muscle of grazing kids. Furthermore, the tenderness and juiciness of kids grazed with concentrate supplementation were similar to those of concentrate-fed kids [29,53], probably due to the similar fat content in kid meat. These results suggest that a supplemented feeding method increases meat tenderness and improves the juiciness of meat better than pasture grazing only.

Overall, pasture grazing reduced the tenderness and juiciness of lamb meat more than stall-feeding, but improved pasture grazing or supplemented grazing was similar or superior to stall-feeding regarding the tenderness and juiciness of the meat. Goat kid meat is more tender but less juicy under pasture grazing than under concentrate feeding, whereas the tenderness and juiciness of kid meat under supplemental grazing are comparable to those under concentrate diets. Moreover, time-limited grazing with supplementary feeding did not affect the juiciness of lamb meat.

**Table 3:** Effects of feeding systems on organoleptic quality attributes of small ruminants.

Items	Animals	Animals ^1^/Duration (Day)	Treatments	Histologic Tissue	Zootechnical Performances ^2^	References
Sheep	5.7-month-old male Norduz lambs	15/84	Concentrate-fed, natural pasture grazing	*Longissimus thoracis*	=WHC	[7]
	37-day-old male Ile de France lambs	16/—	Concentrate-fed, permanent pasture grazing	*Longissimus muscle*	↓Tenderness, Juiciness	[73]
	3-month-old Mongolian lambs	12/180	Concentrate-fed, natural pasture grazing	*Longissimus thoracic*	↑WBSF	[72]
	4-month-old male Tibetan lambs	9/120	Concentrate-fed, desertification grassland grazing	*Longissimus lumborum*	↑WBSF, CL, DL	[70]
	3-month-old male Tan lambs	10/120	Concentrate-fed, natural grassland grazing, time-limited grazing natural grassland supplemented with concentrate, grazing natural grassland supplemented with concentrate	*Longissimus thoracis*	↑CL with pasture grazing; =CL time-limited grazing grassland plus supplementary; =CL with supplemental grazing	[27]
	4-month-old Hulunbui female lambs	22/120	Concentrate-fed, pasture grazing	*Longissimus dorsi*	↑WBSF; ↓WHC; =DL	[36]
	6-month-old male Barbarine lambs	6/90	Stall-feeding, grazing natural pastures improved by lucerne	*Longissimus lumborum*	↑Tenderness, Juiciness	[47]
	3-month-old male Chios lambs	17/60	Concentrate-fed, grazing under olive trees plus supplementation	*Longissimus dorsi*	=CL	[66]
	2-month-old Texel lambs	6/104	Concentrate-fed, grazing *Brachiaria* pastures supplemented with concentrate	*Longissimus lumborum*	=Tenderness, Juiciness	[39]
Goat	Newborn Tibetan goats	25/365	Stall-feeding, natural grass grazing	*Longissimus dorsi*	↓WBSF; ↑WS, CMP	[77]
	4-month-old Cashmere goats	20/104	Concentrate-fed, grazing natural pastures supplemented concentrates	*Longissimus dorsi*	=WL, DL, CL	[29]
	3-month-old Chongming white goats	6/300	Concentrate-fed, grazing pastures supplemented with concentrate	*Longissimus dorsi*	=Tenderness	[53]

= No effect means that no differences were detected among treatments groups (*p* > 0.05). ↑↓ Positive or negative effect means that the treatments showed differences compared to the concentrate-fed/stall-fed group (*p* < 0.05). ^1^ Number of animals per each treatment groups. ^2^ CL, Cooking loss; DL, Drip loss; WBSF, Warner–Bratzler shear force; WHC, Water-holding capacity; WL, water loss; —, none reported.

### 4.3. Meat Flavor of Longissimus Muscle

Flavor is a major factor that determines consumer satisfaction [25,78]. Research shows that the meat of pasture-fed lambs has a superior flavor than concentrate-fed lambs [78]. Moreover, lambs raised on pastures have lower levels of 4-ethyloctanoic acid (EOA), 4-methyloctanoic acid (MOA), and 4-methylnonanoic acid (MNA) and higher levels of 4-methylphenol (MP) compared to concentrate-fed lambs [36] (Table 4). This indicates that the meat flavor of pasture-grazed lambs was better than those of their concentrate-finished counterparts because EOA, MOA, and MNA content influence “mutton” flavor and MP is associated with distinct “pastoral” flavor [79]. Some consumers consider “mutton” and “pastoral” flavors undesirable. The flavors could be associated with a higher crude fat content and carbohydrate presence in concentrate diets [80], promoting the deposition of these compounds. Furthermore, the meat flavor was more pronounced in lambs grazed on natural grassland improved by lucerne than in stall-fed lambs [47]. This could be because lucerne-containing pastures enhance skatole and indole accumulation in the lamb adipose tissue [81]. However, Zhang et al. [70] reported that pasture-grazed lambs had lower benzyl alcohol, 1-heptanol, and alcohol contents and higher decanoic and heptanoic acids contents than concentrate-fed lambs. These findings indicated that pasture grazing reduced the levels of compounds associated with fragrance but increased the levels of compounds related to an unpleasant oily odor. This is because benzyl alcohol and 1-heptanol have strong fragrances, and decanoic acid has an unpleasant oily odor. In [39], da Silva et al. demonstrated that the meat flavor of lambs fed with grazing pasture supplemented with 2.4% body weight of concentrates was similar to those of concentrate-fed lambs. Furthermore, Wang et al. [27] showed that the *longissimus* muscle of the lambs under pasture grazing, grazing with supplementary feeding, or time-limited grazing with supplementary feeding had more flavor (indicated by significantly lower levels of aldehydes and higher levels of alcohols and ketones), compared with concentrate-fed lambs. This could be because of higher levels of aldehydes increasing the production of undesirable odors [27]. Conversely, higher alcohol and ketone contents improve mutton quality and contribute to a distinct flavor of milk or fruits [27]. Consumer preference for lamb flavor is closely related to cultural backgrounds and social-economic conditions [82,83]. A study involving consumers from two European countries (the United Kingdom and Spain) who tasted two types of commercial lamb (stall-fed and pasture-fed) showed that UK consumers preferred pasture-fed lamb with a more intense flavor. In contrast, Spain consumers preferred lamb from intensive feeding systems with a mild flavor [84,85].

The meat of goat kids grazed on pasture contained higher levels of hexanal, nonanal, 1-pentanol, 1-heptanol, 1-octanol, and 2,3-octanedione contents and lower levels of methyl oleate than those maintained under intensive feeding [86]. This implies that pasture grazing enhances the meat flavor of goat kids. Yang et al. [87] also showed that, compared with their stall-fed counterparts, pasture-grazed goat kids had superior flavor in regard to the *longissimus* muscle, which can be attributed to lower isoleucine and leucine contents. These outcomes could be because walking during pasture grazing promotes lipid oxidation in meat, increasing the synthesis of aldehyde and alcohol compounds [87] and resulting in different FA compositions of the meat [88]. In addition, compared with intensive feeding on concentrate diets, grazing pasture with concentrate supplementation exhibited a similar flavor effect on goat kids [53], possibly because of the similar meat IMF content between the two feeding systems [89,90].

Overall, the meat of sheep or goats grazed on pastures has a better meat flavor compared to their stall-fed counterparts. Moreover, the meat flavor of lambs and kids finished under supplemented grazing is comparable to or better than that of their concentrate-fed counterparts. Time-limited grazing with supplementary feeding improved the lamb meat flavor more than concentrate feeding.

**Table 4:** Effects of feeding systems on percentage of volatile flavor compounds of small ruminants.

Items	Animals	Animals ^1^/Duration (Day)	Treatments	Histologic Section	Zootechnical Performances ^2^	References
Sheep	Male Suffolk × ‘Mule’ hybrid lambs	20/—	Concentrate-fed, grassland grazing	*longissimus thoracis et lumborum*	↑flavour	[78]
	4-month-old male Tibetan sheep	9/120	Concentrate-fed, desertification grassland grazing	*longissimus lumborum*	↓Benzyl alcohol, alcohols, 1-Heptanol, ↑Decanoic acid, heptanoic acid	[70]
	4-month-old Hulunbui female lambs	22/120	Concentrate-fed, pasture grazing	*longissimus dorsi*	↓EOA, MOA, MNA; ↑MP; =MI	[36]
	3-month-old male Tan lambs	10/120	Concentrate-fed, natural grassland grazing, time-limited grazing natural grassland supplemented with concentrate, grazing natural grassland supplemented with concentrate	*Longissimus thoracis*	↓Aldehydes; ↑alcohols, ketones with pasture grazing; ↓Aldehydes; ↑alcohols, ketones with time-limited grazing grassland plus supplementary; ↓Aldehydes; ↑alcohols, ketones with supplemental grazing	[27]
	6-month-old male Barbarine lambs	6/90	Stall-feeding, grazing natural pastures improved by lucerne	*Longissimus thoracis*	↑flavour	[47]
	2-month-old Texel lambs	6/104	Concentrate-fed, grazing *Brachiaria* pastures supplemented with concentrate	*Longissimus lumborum*	=flavour	[39]
Goats	Newborn Wulate goats	6/365	Stall-feeding, pasture grazing	*Longissimus dorsi*	↑Hexanal, nonanal, 1-pentanol, 1-heptanol, 1-octanol, 2,3-octanedione; ↓methyl oleate	[86]
	Newborn Black goats	3/365	Stall-feeding, pasture grazing	*Longissimus lumborum*	↑Isoleucine, leucine	[87]
	3-month-old Chongming white goat	6/300	Concentrate-fed, grazing pastures supplemented with concentrate	*Longissimus dorsi*	=flavour	[53]

= No effect means that no differences were detected among treatments groups (*p* > 0.05). ↑↓ Positive or negative effect means that the treatments showed differences compared to the concentrate-fed/stall-fed group (*p* < 0.05). ^1^ Number of animals per each treatment groups. ^2^ MOA, 4-methyloctanoic acid; EOA, 4-ethyloctanoic acid; MNA, 4-methylnonanoic acid; MP, 4-methylphenol; MI, 3-metlylindole; —, none reported.

## 5. Feeding Systems of Small Ruminants Affects the Nutritional Quality Attributes of Meat

### 5.1. Chemical Composition of Longissimus Muscle

Muscle composition, including moisture, protein, IMF, and ash, are among the main attributes of the meat quality of small ruminants, with protein and IMF being the most important properties in meat [27]. Changes in feeding systems affect the meat chemical composition of lambs or kids (Table 5). Regarding sheep lambs, the IMF content in the *longissimus* muscle was lower in lambs grazed on pasture compared with lambs finished on concentrate-based diets [7,27,31,70,72,91]. This could be because the pasture grazing system is characterized by lower energy intake but longer and more intense exercising from navigating the pasture fields than in the stall-feeding system. Another possibility is the differences in rumen microbiota activity between the two feeding systems. It has also been noted that lambs fed on concentrate-based diets have lower rumen acetate/propionate ratios than pasture-grazing lambs, thus resulting in greater IMF deposition [92,93]. However, while investigating the effect of natural grassland grazing and stall-finishing on the chemical composition of the *longissimus* muscle in lambs, Wang et al. [27] found that the protein content was higher, but the IMF content was lower in the natural grassland grazing than in the concentrate feeding. These differences could be due to the higher activity of grazing lambs, which promotes the degradation of carbohydrates and fats but increases the synthesis of proteins. In addition, the meat from lambs raised on grazing pastures plus supplemental feeding or time-limited grazing pastures plus supplemental feeding had a higher protein content and similar fat content to those lambs reared under intensive feeding [31,35,41,55]. This could be because pasture-fed lambs received a greater amount of concentrates, resulting in similar energy intake but greater exercise activity than concentrate-fed animals. These activities prompt the breakdown of carbohydrates and fats and promote protein synthesis.

Regarding goat kids, grazing kids on pastures significantly reduced the IMF but increased the moisture contents compared with stall-feeding, but the protein content in the *longissimus* muscle was comparable between the production systems [56,77]. These findings can be attributed to the lower energy intake and higher energy expenditure under pasture grazing, both of which promote fat catabolism in the muscle tissue and catabolism of muscle fat replaced by water accumulation [70,94,95]. In addition, Dutta et al. [28] and Yu [29] found no significant differences in the protein and IMF contents of the *longissimus* muscle between concentrate-fed and grazed plus supplementation-fed goats. These results indicate that supplemental grazing of kids promotes fat deposition in meat, which, in turn, could contribute to similar fat contents than in concentrate-fed kids. From a human health perspective, meat fat is considered unhealthy in many countries [96,97], and reducing total fat intake could reduce the adverse effects of red meat intake, including obesity and coronary heart disease [91,97]. Thus, leaner meat derived from pasture-only grazed production systems could provide critical health benefits.

Overall, meat from pasture-grazed lambs is lower in protein and IMF contents but similar in the IMF content with those fed on concentrates; however, the protein content is higher in the grazed plus supplementation-fed lambs than in concentrate-fed ones. Meat derived from pasture-grazed goat kids has a similar protein content but lower IMF contents than those fed on concentrates. However, the meat chemical composition was comparable between the grazed plus supplementation-fed and stall-fed kids. Furthermore, the meat of lambs finished with time-limited grazing and concentrate supplementation had higher a protein content and comparable IMF content with their concentrate-fed counterparts.

**Table 5:** Effects of feeding systems on the chemical compositions of the meat of small ruminants.

Items	Animals	Animals ^1^/Duration (Day)	Treatments	Histologic Section	Zootechnical Performances ^2^	References
Sheep	5.7-month-old male Norduz lambs	15/84	Concentrate-fed, natural pasture grazing	*Longissimus thoracis*	=ash; ↑moisture; ↓protein, IMF	[7]
	3-month-old Mongolian lambs	12/180	Concentrate-fed, natural pasture grazing	*Longissimus thoracic*	=protein; ↓IMF, ash	[72]
	3-month-old Sunit sheep	10/270	Stall-feeding, natural pasture grazing	*Longissimus dorsi*	=moisture, protein, ash; ↓IMF	[35]
	4 month-old male Tibetan sheep	9/120	Concentrate-fed, desertification grassland grazing	*Longissimus lumborum*	=moisture, ash; ↓IMF, protein	[70]
	4-month-old Jezersko–Solčava lambs	8/—	Concentrate-fed, mountain pasture grazing	*Longissimus dorsi*	↓IMF	[91]
	3-month-old male Tan lambs	10/120	Concentrate-fed, natural grassland grazing, time-limited grazing natural grassland supplemented with concentrate, grazing natural grassland supplemented with concentrate	*Longissimus thoracis*	=moisture, ash; ↓IMF; ↑protein with pasture grazing =moisture, IMF, ash; ↑protein with time-limited grazing grassland plus supplementary; =moisture, IMF, ash; ↑protein with supplemental grazing	[27]
	3-month-old male Tan lambs	10/120	Concentrate-fed, natural grassland grazing, time-limited grazing natural grassland supplemented with concentrate, grazing natural grassland supplemented with concentrate	*Longissimus dorsi*	↓IMF with pasture grazing =IMF with time-limited grazing grassland plus supplementary; =IMF with supplemental grazing	[31]
	6-month-old male Barbarine lambs	12/240	Concentrate-fed, grazing native pastures supplemented concentrates	*Longissimus thoracis*	=IMF	[55]
Goats	2.9-month-old male Creole goat	60/—	Stall-feeding, irrigated pasture grazing	*Longissimus*	=protein, ash; ↓IMF; ↑moisture	[56]
	Newborn Tibetan goats	25/365	Stall-feeding, natural pasture grazing	*Longissimus dorsi*	=protein; ↓IMF	[77]
	6.3-month-old male Barbari kids	6/110	Concentrate-fed, grazing supplemented with concentrates	*Longissimus dorsi*	=moisture, protein, IMF, ash	[28]
	4-month-old Cashmere goats	20/104	Concentrate-fed, grazing plus supplementation with concentrate	*Longissimus dorsi*	=moisture, protein, IMF, ash	[29]

= No effect means that no differences were detected among treatments groups (*p* > 0.05). ↑↓ Positive or negative effect means that the treatments showed differences compared to the control group (*p* < 0.05). ^1^ Number of animals per each treatment groups. ^2^ IMF, intramuscular fat; —, none reported.

### 5.2. Fatty Acid Content in the Longissimus Muscle

The FA composition of sheep/goat meat is an indicator of meat quality and can signify whether it is safe for human consumption. Among the FAs, SFAs are associated with the development of several diseases [35,52,98]. In contrast, higher levels of n-3 PUFA are beneficial to human health [15,99] because it protects against cardiovascular diseases, certain cancers, and behavioral disorders [100]. Higher levels of n-6 PUFA are deleterious for human health because it predisposes consumers to coronary diseases [99,101,102].

Studies showed that the SFAs in the *longissimus* muscle of pasture grazing or supplemental grazing lambs were comparable to or lower than that of stall-fed lambs (Table 6) [31,35,91,98,103,104]. In contrast, levels of PUFA and n-3 PUFA were comparable or higher among pasture-grazing and supplemental-grazing lambs compared with stall-feeding lambs [31,35,91,98,103,104]. This could be due to the differences in lipid content and fatty acid composition in the intake feeds and diets [105,106], which modify the ruminal environment and microbial species composition [107,108,109], subsequently affecting the FA composition of the lamb. These findings suggest that meat from pasture-grazed or supplemented grazing lambs contains higher contents of beneficial FAs. However, Dervish et al. [110] demonstrated that lambs grazing on pasture had higher n-3 PUFA and CLA levels in the *longissimus* muscle than those fed the same hay pasture. However, no differences were observed in the levels of other FAs between the two feeding systems, probably because there were no extreme differences in the diets. Additionally, SFA and MUFA levels were similar or lower in the group fed via time-limited grazing (4 h) on natural grassland with concentrate supplements compared with concentrate feeding [103]. However, n-3 PUFA levels were higher in lambs under time-limited grazing on natural grassland plus supplemental concentrates [103]. These findings indicate that reducing grazing times and incorporating concentrate supplements can also improve lamb meat quality. These results can be explained by the fact that grazing increases forage intake but reduces concentrate intake, which, in turn, increases the levels of n-3 PUFA and decrease SFA levels. The SFA levels in the *longissimus* muscle of goat kids are equal to or lower than kids grazed on pasture and those under stall-feeding conditions [52,87]. However, the levels of PUFA, n-3 PUFA, and n-6 PUFA were comparable or higher in kids grazed on pasture [52,87]. In addition, the levels of SFA, PUFA, n-3 PUFA, and n-6 PUFA were comparable between the meat of kids that are grazed/supplementation-fed and those fed concentrate diets [29,111]. The MUFA levels were comparable or lower in grazed kids [29,111]. These findings suggest that supplementing grazing with concentrates did not improve the FA composition of meat.

Higher levels of lauric (C12:0), myristic (C14:0), and palmitic (C16:0) acids increase the risk of developing cardiovascular diseases in humans [7], whereas oleic acid (C18:1n9), the highest component of MUFA, reduces the cholesterol content in human blood, lowering the atherogenic potential [7,112,113]. Moreover, linoleic (C18:2n-6), α-linolenic (ALA, C18:3n-3), γ-linolenic (GLA, C18:3n-6), arachidonic (C20:4n-6), docosapentaenoic (DPA, C22:5n-3), eicosapentaenoic (EPA, C20:5n-3), docosahexaenoic (DHA, C22:6n-3), and conjugated linoleic acids (CLAs) have anticarcinogenic properties and prevent several pathologies, such as chronic or cardiovascular diseases [114,115,116]. Regarding sheep lambs, the C18:2n-6, GLA, ALA, C20:4n-6, DHA, and CLAs were the same or higher in the *longissimus* muscle of lambs grazed on pasture or grazed plus concentrate supplements than those fed on concentrate diets [31,35,91,98,103,104]. However, C12:0, C14:0, C16:0, and C18:1n-9 contents were the same or lower, while EPA and DPA contents were higher in lambs grazed on pasture only or pasture plus concentrate supplements [31,35,91,98,103,104]. These findings suggest that the meat from lambs fed on pastures only or grazed with supplemental concentrates has more beneficial FA content but lower harmful FA content. These results could be due to the fact that higher energy intake promotes SFA synthesis and increases the C18:3n3 ratio in herbage, while higher forage intake promotes the growth of fibrolytic microorganisms responsible for the rumen hydrogenation process, increasing the production of CLAs. Regarding goat kids, C18:2n-6, ALA, GLA, and DHA were equal or higher, while C14:0 and C16:0 contents were lower in goat kid meat grazed only on pasture than in those fed on concentrates [52,87]. These findings indicate that the meat of kids grazed on pasture has higher levels of healthy FAs than those fed with concentrates. Therefore, consumers may prefer meat from kids fed more on grass instead of concentrates. However, there was no significant difference in FA composition between grazing plus supplemental-fed and concentrate-fed kids [29].

From a scientific point of view, and the perspective of consumers, the recommended ratio of PUFA/SFA should be higher than 0.45, whereas the levels of n-6/n-3 PUFA should not exceed 4 [104,113,117,118]. The *longissimus* muscle of pasture-grazing (average 0.50 and 2.73, respectively) or supplemented grazing (average 0.48 and 3.90, respectively) lambs had a similar or higher PUFA/SFA ratio and a similar or lower ratio of n-6/n-3 PUFA compared with stall-feeding animals (average 0.39 and 10.20, respectively) [31,35,55,98,103,104,113]. These effects could be due to the predominance of α-linolenic and linoleic acids (the parent molecules of the n-3 PUFA and n-6 PUFA families) in forages and grains, respectively [10,119,120]. Moreover, the meat of lambs reared on limited-time grazing (4 h) on natural grassland plus concentrate supplement (average 0.31 and 6.25, respectively) had higher levels of PUFA/SFAs and lower n-6/n-3 PUFA ratios compared with those fed on concentrates alone (average 0.23 and 20.00, respectively) [31,103]. These results show that, compared with the stall-feeding system, the PUFA/SFA and n-6/n-3 PUFA ratios of the *longissimus* muscle of lambs reared by pasture-grazing or grazing supplemented with concentrates could be close to the standard PUFA/SFA and n-6/n-3 PUFA ratios. This indicated that the FA composition observed in the two feeding systems was reasonable. Goat kids grazed on pasture (average 4.07) had lower levels of n-6/n-3 PUFA in the *longissimus* muscle than those maintained under intensive feeding (average 7.89) [52,87], suggesting that kids fed exclusively on pastures have a desirable FA composition. Furthermore, the PUFA/SFA and n-6/n-3 PUFA contents in the *longissimus* muscle of kids on supplemented grazing (average 0.38 and 2.48, respectively) were comparable to those maintained under intensive feeding (average 0.37 and 2.47, respectively) [29,111]. This suggested that supplemented grazing did not improve FA composition in meat more than the intensive feeding concentrates.

Overall, compared to stall-fed or concentrate-fed lambs, lambs grazing on pasture alone or pasture with supplemental concentrate feeding yield a higher composition of beneficial FA but a lower composition of SFAs (particularly C12:0, C14:0, and C16:0). These lambs also have higher levels of n-3 PUFAs (particularly EPA and DPA) and general standard PUFA/SFA and n-6/n-3 PUFA ratios. The meat of kids fed on pasture alone has higher levels of beneficial FA, lower levels of SFA (e.g., C14:0 and C16:0), higher n-3 PUFA levels (e.g., C18:2n-6, ALA, GLA, and DHA), and general standard PUFA/SFA and n-6/n-3 PUFA ratios. However, kids under grazing combined with supplemental concentrate feeding produce meat with FA content comparable to those fed on concentrates alone. Furthermore, lambs/kids under time-limited grazing with supplementary concentrates produce meat enriched with higher n-3 PUFA contents (e.g., ALA, and EPA) and general standard n-6/n-3 PUFA ratios than their concentrate-fed counterparts.

**Table 6:** Effects of feeding systems on the fatty acid content in the meat of small ruminants.

Items	Animals	Animals ^1^/Duration (Day)	Treatments	Histologic Section	Zootechnical Performances ^2^	References
Sheep	3-month-old Sunit sheep	10/270	Stall-feeding, natural pasture grazing	*Longissimus dorsi*	=SFA, MUFA, PUFA, PUFA/SFA; ↑ n-3 PUFA; ↓ n-6 PUFA, n-6/n-3 =C12:0, C14:0, C16:0, C18:1n-9; ↑ C18:3n-3, C20:5n-3, C22:6n-3, CLAs; ↓ C18:2n-6, C20:4n-6	[35]
	Newborn Churra Tensina sheep	24/—	Stall-feeding, natural pasture grazing	*Longissimus dorsi*	=SFA, MUFA, PUFA, n-6 PUFA, PUFA/SFA, n-6/n-3; ↑ n-3 PUFA =C12:0, C14:0, C16:0, C18:1n-9, C20:5n-3, C22:6n-3; ↑ CLA	[110]
	4-month-old Tan sheep	12/60	Concentrate-fed, pasture grazing	*Longissimus thoracis*	=MUFA; ↑ PUFA, n-3PUFA, n-6 PUFA; ↓SFA, n-6/n-3 =C12:0, C14:0, C18:3n6; ↑ C18:2n-6, C18:3n-3, C20:4n-6, C20:5n-3, C22:6n-3; ↓ C16:0, C18:1n-9	[98]
	0.5-month-old Ile de France lambs	20/93	concentrate-fed, natural pasture grazing	*Longissimus dorsi*	=MUFA; ↑ PUFA, n-6 PUFA, n-3 PUFA, PUFA/SFA; ↓ SFA, n-6/n-3 ↑ C18:2n-6, C18:3n-3, C18:3n-6, C20:4n-6, C20:5n-3, C22:5n-3, C22:6n-3, CLAs; ↓ C12:0, C14:0, C16:0, C18:1n-9	[113]
	3-month-old male Tan lambs	10/120	Concentrate-fed, natural grassland grazing, time-limited grazing natural grassland supplemented with concentrate, grazing natural grassland supplemented with concentrate	*Longissimus dorsi*	=SFA; ↑ PUFA, n-3 PUFA, n-6 PUFA; ↓ MUFA, n-6/n-3 =C20:4n6, CLAs; ↑ C18:2n-6, C18:3n-6, C18:3n-3, C20:5n-3; ↓ C12:0, C14:0, C16:0, C18:1n-9 with pasture grazing	[31]
					=SFA, MUFA; ↑ PUFA, n-3 PUFA, n-6 PUFA; ↓ n-6/n-3 =C18:1n-9, C20:4n-6, CLAs; ↑ C18:3n-6, C18:3n-3, C20:5n-3; ↓ C12:0, C14:0, C16:0 with supplemental grazing	
					=SFA, MUFA, PUFA, n-6 PUFA; ↑ n-3 PUFA; ↓ n-6/n-3 =C12:0, C14:0, C16:0, C18:1n-9, C18:3n-6, C20:4n-6; ↑ C18:3n-3, C20:5n-3 with time-limited grazing grassland plus supplementary	
	6-month-old male Barbarine lambs	12/240	Concentrate-fed, grazing native pastures supplemented concentrates	*Longissimus thoracis*	=SFA, MUFA, PUFA, n-6 PUFA, n-3 PUFA; n-6/n-3; ↑ PUFA/SFA =C14:0, C16:0, C18:1n-9, C18:2n-6, C18:3n-3, C20:4n-6; ↑ C12:0, C20:5n-3, C22:6n-3; ↓ C18:3n-6	[55]
	4-month-old male Tan lambs	13/83	Concentrate-fed, natural grassland grazing, time-limited grazing natural grassland plus supplementation	*Longissimus dorsi*	↑PUFA, PUFA/SFA; ↓SFA, MUFA with pasture grazing	[103]
					=PUFA; ↑ PUFA/SFA; ↓SFA, MUFA with time-limited grazing grassland plus supplementary	
	5.4-month-old male Romane lambs	12/101	Concentrate-fed, grazed alfalfa grazing, grazed alfalfa plus supplementation	*Longissimus thoracis*	=SFA, MUFA, n-6 PUFA, PUFA/SFA; ↑ PUFA, n-3 PUFA; ↓ n-6/n-3 =C12:0, C14:0, C16:0; C18:1n-9; ↑ C18:3n-3, C20:5n-3, C22:5n-3, C22:6n-3, CLAs with pasture grazing	[104]
					=SFA, PUFA; ↑ n-3 PUFA, PUFA/SFA; ↓ MUFA, n-6 PUFA, n-6/n-3 =C12:0, C14:0, C16:0; ↑ C18:3n3, C20:5n-3, C22:5n-3, C22:6n-3, CLAs; ↓ C18:1n-9 with supplemental grazing	
Goats	4-month-old Albas White Cashmere kids	30/60	Concentrate-fed, natural pasture grazing	*Longissimus thoracis*	=SFA, n-6 PUFA; ↑ PUFA, n-3 PUFA, PUFA/SFA; ↓ MUFA, n-6/n-3 =C12:0, C20:4n-6; ↑ C18:2n-6, C18:3n-3, C18:3n-6, C20:5n-3, C22:6n-3; ↓ C14:0, C16:0	[52]
	Newborn black kids	3/365	Stall-feeding, pasture grazing	*Longissimus lumborum*	=MUFA, PUFA, n-3 PUFA; ↑ n-6 PUFA; ↓ SFA =C18:1n-9; C18:2n-6, C18:3n-3, C18:3n-6, C20:4n-6; ↓ C12:0, C14:0, C16:0, C20:5n-3	[87]
	4-month-old Cashmere kids	20/114	Concentrate-fed, grazing natural pastures supplemented concentrates	*Longissimus thoracis*	=SFA, PUFA, n-3 PUFA, n-6 PUFA, PUFA/SFA, n-6/n-3;↓ MUFA =C12:0, C14:0, C16:0, C18:2n-6, C18:3n-6, C20:4n-6, C18:3n-3, C20:5n3, ↓ C18:1n-9	[29]
	3-month-old Chongming white kids	14/—	Concentrate-fed, grazing pastures plus supplemented concentrate	*Longissimus dorsi*	=SFA, MUFA, PUFA, PUFA/SFA =C14:0, C16:0; ↑ CLA	[111]

= No effect means that no differences were detected among treatments groups (*p* > 0.05). ↑↓ Positive or negative effect means that the treatments showed differences compared to the control group (*p* < 0.05). ^1^ Number of animals per each treatment groups. ^2^ CLAs, conjugated linoleic acids; MUFA, monounsaturated fatty acids; N.R., not reported; PUFA, polyunsaturated fatty acids; SFA, saturated fatty acids; —, none reported.

## 6. Conclusions

Feeding systems affect the growth rate, carcass traits, and meat quality attributes of small ruminants. The effect of feeding system changes on performance and carcass traits were the same for sheep and goats, but their effects on meat quality varied between the two species. Compared with stall-feeding or concentrate feeding, lambs/kids grazed on pasture have reduced ADG and carcass attributes (e.g., lower SLW, HCW, CCW, DP), but those grazed on high-quality pastures have higher SLW and growth rates. Moreover, compared with concentrate-feeding, supplemented grazing results in comparable ADG and carcass yield. Time-limited grazing combined with supplementary feeding improves the HCW of lambs. Compared with stall-fed lambs, pasture grazing lambs have improved meat flavor and healthy FA composition but reduced meat color, tenderness, juiciness, protein, and IMF content. Furthermore, supplementing grazing lambs with concentrates improves the protein content and healthy FA composition of lamb meat than concentrate feeding. A similar trend was observed for the organoleptic meat properties between supplementing grazing and stall-feeding lambs. Meat from pasture-grazing goat kids has a dull color, reduced juiciness and IMF content, greater tenderness, enhanced flavor, and superior healthy FA composition than those fed on concentrates. Moreover, kids grazed on concentrate supplements show improved meat color, and there is little effect on other meat quality attributes compared with concentrate feeding. Time-limited grazing with supplements improves the HCW, protein content, and healthy FA composition of lamb meat. However, the effects of time-limited grazing in different seasons and types of improved pasture (supplemented with different compositions of concentrates) on the growth rate, carcass attributes, and meat quality of ovine and caprine species should be explored in the future.

## Data Availability

No new data were analyzed or created in this study. Data available provided in this study is public and available for download.
